# Supplementary material for: The genomic landscape of Mexican Indigenous populations brings insights into the peopling of the Americas
Source: Nat Commun. 2021 Oct 12;12:5942. doi: 10.1038/s41467-021-26188-w (PMC8511047; doi:10.1038/s41467-021-26188-w)
Supplement: Supplementary file 1 — Supplementary Information [file 41467_2021_26188_MOESM1_ESM.pdf]

The genomic landscape of Mexican Indigenous populations brings insights into the peopling of the Americas

Humberto García-Ortiz[1*¥], Francisco Barajas-Olmos[1¥], Cecilia Contreras-Cubas[1¥], Miguel Ángel Cid-Soto[1], Emilio J. Córdova[1], Federico Centeno-Cruz[1], Elvia Mendoza-Caamal[1], Isabel Cicerón-Arellano[1], Marlen Flores-Huacuja[1], Paulina Baca[1], Deborah A. Bolnick[2], Meradeth Snow[3], Silvia Esperanza Flores-Martínez[4], Rocío Ortíz-López[5], Austin W. Reynolds[6], Antonio Blanchet[1], Mirna Morales-Marín[1], Rafael Velázquez-Cruz[1], Aleksandar David Kostic[7], Carlos Galaviz-Hernández[8], Alejandra Guadalupe García-Zapién[9], José Concepción Jiménez-López[10†], Guadalupe León-Reyes[1], Eva Gabriela Salas-Bautista[10], Blanca Patricia Lazalde-Ramos[11], Juan Luis Jiménez-Ruíz[1], Guadalupe Salas-Martínez[1], Jazmín Ramos-Madrigal[12], Elaheh Mirzaeicheshmeh[1], Yolanda Saldaña-Alvarez[1], María del Carmen Abrahantes-Pérez[1], Francisco Loeza-Becerra[13], Raúl Mojica-Espinosa[1], Federico Sánchez-Quinto[1], Héctor Rangel-Villalobos[14], Martha Sosa-Macías[8], José Sánchez-Corona[4†], Augusto Rojas-Martínez[5], Angélica Martínez-Hernández[1], Lorena Orozco[1*].

*Correspondence to: lorozco@inmegen.gob.mx; hgarcia@inmegen.gob.mx.

¥These authors contributed equal to this work.

This PDF file includes:

Supplementary Tables 1 to 5.
Supplementary Figs. 1 to 18.

Supplementary table 1. Mexican Indigenous populations Included in the present study.

Ethnic Group	N	N after QC	Linguistic Family†	Main Geographic/Cultural Area	Latitude	Longitude	Source	Genotyping plataform
Cucapa	7	7	Cochimi-yumana	Aridoamerica	32.27	-114.46	This Study	Affymetrix 6.0
Kiliwa	1	1			32.11	-116.41	This Study	Affymetrix 6.0
Kuahl	1	1			31.52	-116.36	This Study	Affymetrix 6.0
Kumiai	3	3			31.52	-116.35	This Study	Affymetrix 6.0
Paipai	2	2			32.11	-116.41	This Study	Affymetrix 6.0
Seri	10	10	Seri		29.01	-112.11	This Study	Affymetrix 6.0
Seri (2)*	24	16			29.00	-112.15	2	Affymetrix 6.0
Papago	8	8	Yuto-nahua		30.43	-111.50	This Study	Affymetrix 6.0
Pima (8)*	33	32			29.30	-108.80	8	Illumina 650 Y array
Pima	1	1			28.22	-108.55	This Study	Affymetrix 6.0
Yaqui (8)*	1	1			28.00	-113.00	8	Illumina 610-Quad array
Tarahumara (2)*	24	22			27.75	-107.17	2	Affymetrix 6.0
Yaqui	10	10			27.55	-110.54	This Study	Affymetrix 6.0
Tarahumara	56	52			27.22	-108.17	This Study	Affymetrix 6.0
Mayo	10	10			27.04	-109.29	This Study	Affymetrix 6.0
Guarijio	10	10			27.01	-108.56	This Study	Affymetrix 6.0
Huichol	11	11	Yuto-nahua	Mesoamerica	23.28	-104.23	This Study	Affymetrix 6.0
Mexicanero	10	10			23.28	-104.23	This Study	Affymetrix 6.0
Tepehuano	12	12			23.20	-104.50	This Study	Affymetrix 6.0
Tepehuano (9)*	20	14			23.20	-104.50	9	Affymetrix 500K
Cora	11	11			22.07	-105.12	This Study	Affymetrix 6.0
Huichol (2)*	24	22			21.17	-104.08	2	Affymetrix 6.0
Nahuatl_CDMX	16	16			19.26	-99.08	This Study	Affymetrix 6.0/Illumina OMNI 2.5
Nahuatl_Gro	1	1			17.43	-98.56	This Study	Affymetrix 6.0
Nahuatl_Jal (2)*	20	20			19.50	-103.50	2	Affymetrix 6.0
Nahuatl_Mex	27	27			19.30	-99.43	This Study	Affymetrix 6.0/Illumina OMNI 2.5
Nahuatl_Mor	10	10			18.51	-99.19	This Study	Affymetrix 6.0
Nahuatl_Pue	10	10			19.49	-97.39	This Study	Affymetrix 6.0
Nahuatl_Pue (2)*	41	17			19.93	-97.62	2	Affymetrix 6.0
Nahuatl_SLP	12	10			21.25	-98.44	This Study	Affymetrix 6.0/Illumina OMNI 2.5
Nahuatl_Trios (2)*	23	21			19.97	-97.62	2	Affymetrix 6.0
Nahuatl_Ver	4	3			18.13	-94.52	This Study	Affymetrix 6.0
Purepecha	16	16	Tarasca		19.15	-102.22	This Study	Affymetrix 6.0/Illumina OMNI 2.5
Purepecha (2)*	23	21			19.75	-101.50	2	Affymetrix 6.0
Purepecha (8)*	1	1			19.00	-101.50	8	Illumina 610-Quad array
Tepehua	1	1	Totonaco-tepehua		20.37	-98.12	This Study	Affymetrix 6.0
Totonaco (2)*	24	21			20.00	-97.80	2	Affymetrix 6.0
Totonaco_Pue	11	11			20.43	-97.51	This Study	Affymetrix 6.0
Totonaco_Ver	10	10			20.15	-97.39	This Study	Affymetrix 6.0
Amuzgo	2	2	Oto-mangue		16.41	-98.24	This Study	Affymetrix 6.0
Chatino	2	2			16.13	-97.16	This Study	Affymetrix 6.0
Chichimeca	1	1			21.17	-100.31	This Study	Affymetrix 6.0
Chinanteco	10	10			18.04	-96.23	This Study	Affymetrix 6.0
Chocholteco	6	6			16.57	-96.28	This Study	Affymetrix 6.0
Cuicateco	3	3			17.20	-97.53	This Study	Affymetrix 6.0
Ixcateco	1	1			17.51	-97.11	This Study	Affymetrix 6.0
Matlaltzinca	10	10			19.10	-99.54	This Study	Affymetrix 6.0
Mazahua	23	17			19.17	-99.38	This Study	Affymetrix 6.0/Illumina OMNI 2.5
Mazateco (2)*	17	15			18.33	-96.33	2	Affymetrix 6.0
Mazateco_Oax	14	12			18.22	-96.44	This Study	Affymetrix 6.0
Mazateco_Pue	10	10			18.16	-96.59	This Study	Affymetrix 6.0
Mixteco (8)*	5	5			17.00	-97.00	8	Illumina 610-Quad array
Mixteco_alto	12	12			17.32	-97.43	This Study	Affymetrix 6.0
Mixteco_costa	10	10			16.08	-97.22	This Study	Affymetrix 6.0
Otomi_Hgo	50	47			20.29	-99.13	This Study	Affymetrix 6.0
Otomi_Mex	15	15			19.28	-99.32	This Study	Affymetrix 6.0/Illumina OMNI 2.5
Otomi_Qro	2	2			20.11	-100.08	This Study	Affymetrix 6.0
Pame	10	10			22.55	-100.55	This Study	Affymetrix 6.0
Popoloca	2	2			18.35	-97.55	This Study	Affymetrix 6.0
Tlahuica	10	10			19.00	-99.23	This Study	Affymetrix 6.0
Tlapaneco	2	2			16.58	-98.40	This Study	Affymetrix 6.0
Triqui	3	3			17.17	-96.53	This Study	Affymetrix 6.0
Triqui (2)*	24	22			17.28	-97.95	2	Affymetrix 6.0
Zapoteco	14	13			16.57	-96.28	This Study	Affymetrix 6.0
Zapoteco_N (9)*	21	21			17.41	-96.69	9	Affymetrix 500K
Zapoteco_S (2)*	24	18			17.23	-96.23	2	Affymetrix 6.0
Chontal_Oax	11	11	Chontal de Oaxaca		16.03	-95.67	This Study	Affymetrix 6.0/Illumina OMNI 2.5
Huave	10	10	Huave		16.34	-94.52	This Study	Affymetrix 6.0
Mixe	10	10	Mixe-zoque		16.57	-95.05	This Study	Affymetrix 6.0
Mixe (8)*	17	17			17.00	-96.00	8	Illumina 610-Quad array
Popoluca_Sierra	10	10			18.14	-94.52	This Study	Affymetrix 6.0
Zoque	20	20			17.11	-93.03	This Study	Affymetrix 6.0
Awakateko	1	1	Maya		19.30	-90.30	This Study	Affymetrix 6.0
Chol	10	10			17.18	-92.26	This Study	Affymetrix 6.0
Chontal_Tab	2	2			18.10	-93.01	This Study	Affymetrix 6.0
Chuj	10	10			15.23	-92.11	This Study	Affymetrix 6.0
Huasteco	10	10			21.37	-99.01	This Study	Affymetrix 6.0
Ixil	1	1			19.29	-89.59	This Study	Affymetrix 6.0
Poptiacalteco	10	10			16.30	-92.25	This Study	Affymetrix 6.0
Kanjobal	10	10			15.23	-92.13	This Study	Affymetrix 6.0
Kaqchikel	10	10			15.23	-92.11	This Study	Affymetrix 6.0
Kekchi	2	2			19.20	-90.43	This Study	Affymetrix 6.0
Lacandon	11	11			16.54	-92.05	This Study	Affymetrix 6.0
Lacandon (2)*	22	8			16.75	-91.25	2	Affymetrix 6.0
Mam	12	12			15.30	-92.13	This Study	Affymetrix 6.0
Maya_Camp	2	2			19.60	-90.40	This Study	Affymetrix 6.0/Illumina OMNI 2.5
Maya_Camp (9)*	45	27			20.37	-90.05	9	Affymetrix 500K
Maya_QR (2)*	18	15			19.58	-88.58	2	Affymetrix 6.0
Maya_Yuc	20	20			21.08	-88.06	This Study	Affymetrix 6.0
Mocho	10	10			15.22	-92.14	This Study	Affymetrix 6.0
Tojolabal	10	10			15.24	-92.12	This Study	Affymetrix 6.0
Tojolabal (2)*	21	15			16.50	-92.00	2	Affymetrix 6.0
Tzeltal	15	14			16.54	-92.05	This Study	Affymetrix 6.0
Tzotzil	16	16			17.04	-92.51	This Study	Affymetrix 6.0
Tzotzil (2)*	21	18			16.83	-92.67	2	Affymetrix 6.0

* Number between brackets represent the study source.
† Linguistic Clasification was done based on INALI classification.

Supplementary table 2. Populations included in demographic reconstructions.

Population	Sample Size	Study Source
Seri	26	This Study/Moreno-Estrada, et al.
Mayo	10	This Study
Yaqui	10	This Study
Guarijio	10	This Study
Tarahumara	74	This Study/Moreno-Estrada, et al.
Huichol	35	This Study/Moreno-Estrada, et al.
Cora	11	This Study
Mexicanero	10	This Study
Tepehuano	12	This Study
Nahuatl_Jal	20	Moreno-Estrada, et al.
Purepecha	37	This Study
Pame	10	This Study
Otomi_Hgo	47	This Study
Nahuatl_Mor	10	This Study
Matlaltzinca	10	This Study
Mazahua	17	This Study
Tlahuica	10	This Study
Huasteco	10	This Study
Totonaco_Ver	10	This Study
Totonaco_Pue	11	This Study
Totonaco	21	Moreno-Estrada, et al.
Nahuatl_Pue	27	This Study/Moreno-Estrada, et al.
Chinanteco	10	This Study
Mixteco_alto	12	This Study
Mixteco_costa	10	This Study
Mazateco_Pue	10	This Study
Mazateco	15	Moreno-Estrada, et al.
Mazateco_Oax	12	This Study
Triqui	24	This Study/Moreno-Estrada, et al.
Zapoteco	25	This Study/Moreno-Estrada, et al.
Mixe	10	This Study
Chontal_Oax	10	This Study
Huave	10	This Study
Popoluca_Sierra	10	This Study
Chol	10	This Study
Zoque	20	This Study
Maya_Yuc	20	This Study
Maya_QR	15	Moreno-Estrada, et al.
Tzeltal	14	This Study
Poptijacalteco	10	This Study
Kaqchiquel	10	This Study
Mame	12	This Study
Mocho	10	This Study
Tzotzil	34	This Study/Moreno-Estrada, et al.
Chuj	10	This Study
Kanjobal	10	This Study
Tojolabal	25	This Study/Moreno-Estrada, et al.
Lacandon	19	This Study/Moreno-Estrada, et al.

Supplementary table 3. Long-term effective population size by population.

Population*	Ne	CI
Seri	984	909 - 1092
Mayo	2508	1527 - 3328
Yaqui	2335	1193 - 4193
Guarijio	1832	1528 - 2138
Tarahumara	2444	2222 - 2660
Huichol	2043	1933 - 2189
Cora	2162	1833 - 2721
Mexicanero	1825	1548 - 2140
Tepehuano	2448	2062 - 2835
Nahuatl_Jal(2)	2829	2589 - 3440
Purepecha	3331	3032 - 3694
Pame	2542	2187 - 2850
Otomi_Hgo	2917	2747 - 3239
Nahuatl_Mor	2193	1817 - 2396
Matlaltzinca	2433	2135 - 2689
Mazahua	2711	2473 - 3014
Tlahuica	1993	1679 - 3220
Huasteco	2594	2384 - 2939
Totonaco_Ver	3002	2617 - 3425
Totonaco_Pue	2523	2283 - 3066
Totonaco(2)	2418	2227 - 2825
Nahuatl_Pue	3212	2897 - 3933
Chinanteco	2598	2197 - 2977
Mixteco_alto	2797	2390 - 3714
Mixteco_costa	2507	2225 - 2673
Mazateco_Pue	2642	2255 - 2899
Mazateco(2)	2669	2422 - 2947
Mazateco_Oax	2160	1856 - 2678
Triqui	2032	1870 - 2369
Zapoteco	2805	2563 - 3064
Mixe	2527	2256 - 3198
Chontal_Oax	2722	1614 - 3584
Huave	2062	1550 - 2938
Popoluca_Sierra	2199	1930 - 2608
Chol	2621	2111 - 3026
Zoque	2635	2465 - 2818
Maya_Yuc	3291	3069 - 3685
Maya_QR(2)	3079	2842 - 3428
Tzeltal	2678	2337 - 2880
Jakalteko	2094	1336 - 3105
Kaqchiquel	2599	2239 - 2849
Mame	2577	2229 - 2924
Mocho	2371	2027 - 2862
Tzotzil	2749	2519 - 2966
Chuj	2353	2120 - 2550
Kanjobal	2290	2072 - 2662
Tojolabal	2330	2077 - 2617
Lacandon	1593	1372 - 1752

* Number between brackets represent the study source.

Supplementary table 4. Mexican Native american from MAIS cohort have at least 0.99 indigenous ancestry.

Population	N	Study source
Awakateko	1	This Study
Chinanteco	7	
Chocholteco	1	
Chol	6	
Chontal_Oax	1	
Chuj	10	
Cora	3	
Cucapa	2	
Cuicateco	1	
Guarijio	10	
Huasteco	9	
Huave	1	
Huichol	10	
Ixil	1	
Jakalteko	2	
Kanjobal	10	
Kaqchiquel	2	
Kekchi	2	
Lacandon	7	
Mame	6	
Matlaltzinca	7	
Maya_Camp	1	
Maya_Yuc	11	
Mazahua	5	
Mazateco_Oax	5	
Mazateco_Pue	9	
Mexicanero	10	
Mixe	6	
Mixteco_alto	11	
Mixteco_costa	10	
Mocho	4	
Nahuatl_Mor	10	
Nahuatl_Pue	3	
Nahuatl_SLP	7	
Nahuatl_Ver	2	
Otomi_Hgo	22	
Pame	4	
Pima	1	
Popoloca	2	
Popoluca_Sierra	6	
Seri	10	
Tarahumara	29	
Tepehuano	4	
Tlapaneco	2	
Tojolabal	4	
Totonaco_Pue	11	
Totonaco_Ver	5	
Triqui	3	
Tzeltal	10	
Tzotzil	13	
Zapoteco	6	

Supplementary table 5. Ancient and Modern genomes used for Treemix and D-statistics simulations.

Population	N	Study source
USR1	1	Moreno-Mayar, J. V. et al. 2018
Anzick-1	1	Rasmussen, M. et al. 2018
Saqqaq	1	Rasmussen, M. et al. 2010
Lucier	4	Scheib, C. L. et al. 2018
Alaskan Athabaskan	1	
Aleut	2	Mallick, S. et al, 2016
Cree	2	
Chipewyan	2	
Karitiana	2	
Quechua	3	
Aymara	1	Raghavan, M. et al. 2015

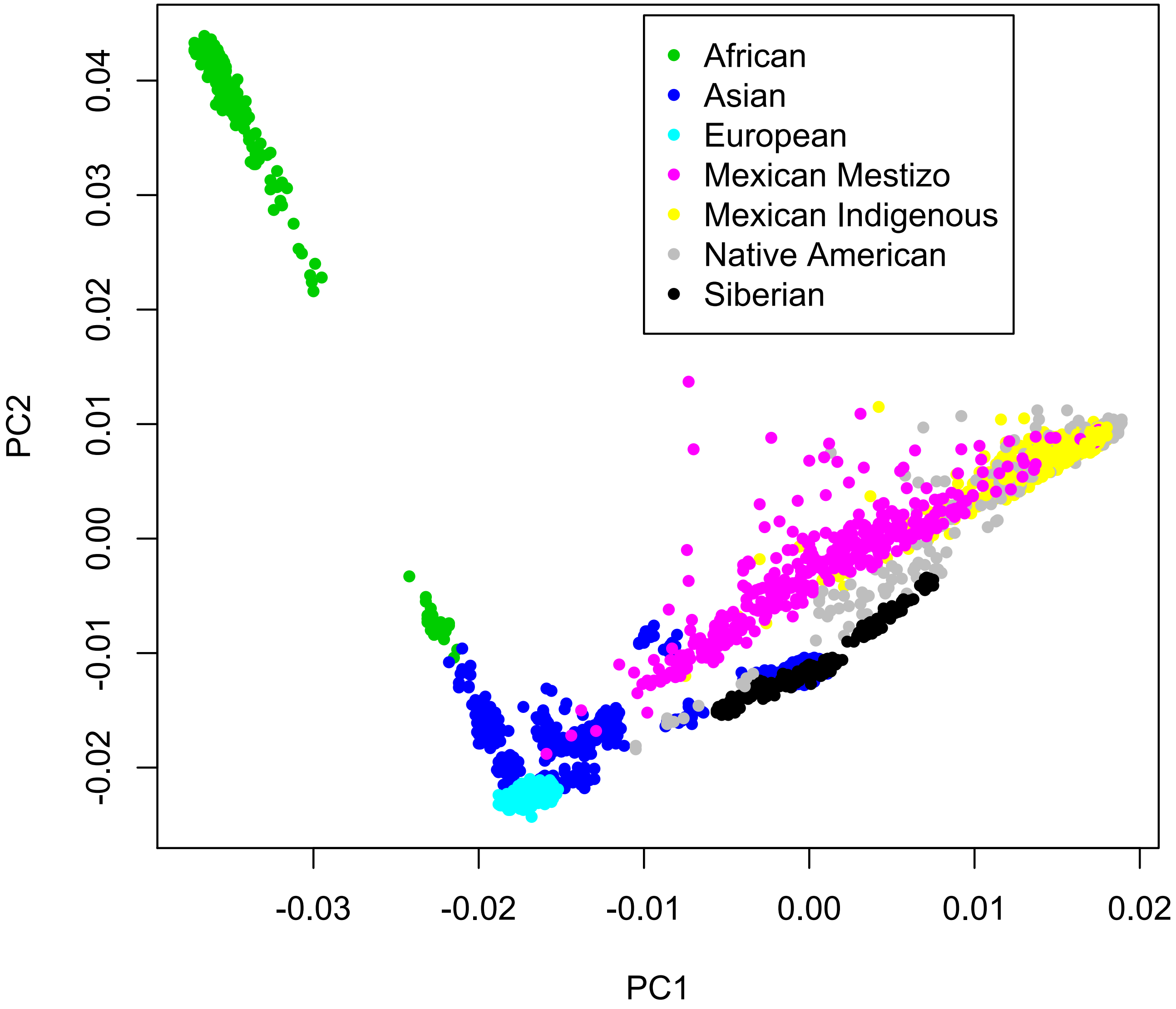

Supplementary Figure 1. Principal component analysis performed in worldwide populations. Projection of top two principal components shows that Mexican Indigenous populations can be grouped with other Native populations from the Americas.

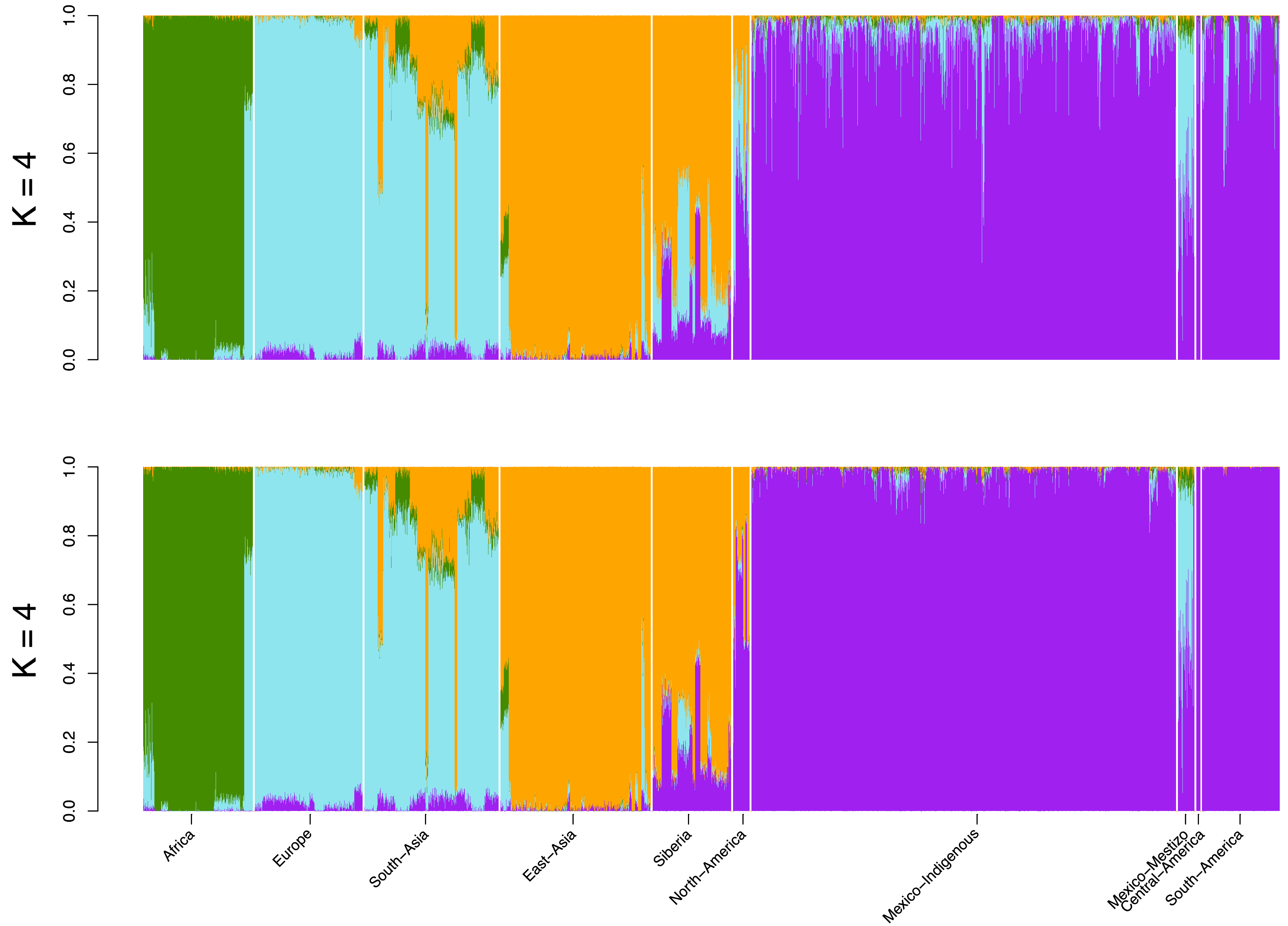

Supplementary Figure 2. Admixture inference assuming K = 4 clusters. The inference of ancestry proportions in raw data set (upper panel) and ancestry masked data set (down panel) show the effectiveness of the local ancestry estimation to mask no Native American ancestry tracks. Purple colored bars represent the proportion of assumed Native American ancestry in each sample.

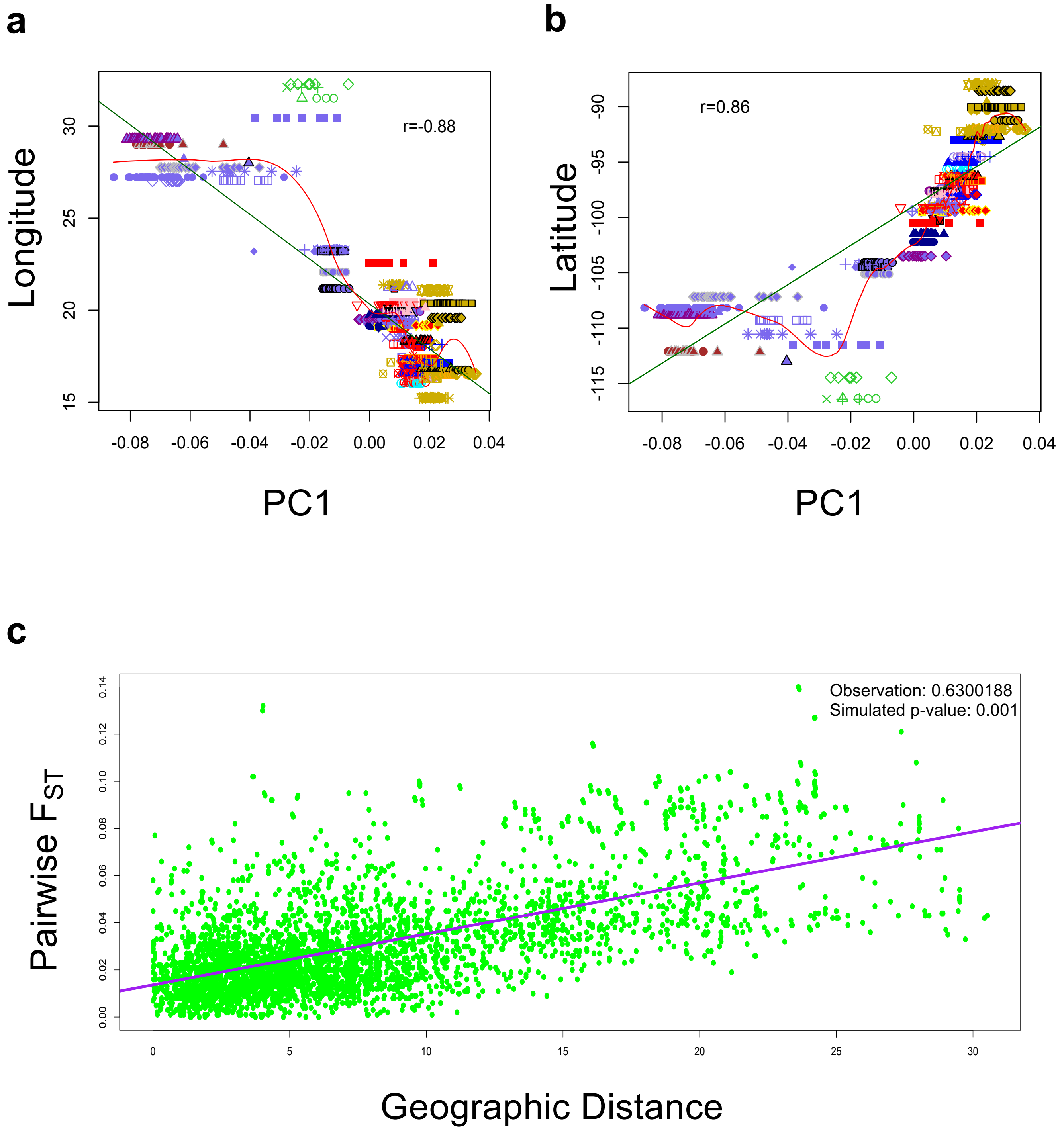

Supplementary Figure 3. Genetic diversity of Mexican Indigenous populations is influenced by geographic location. Correlation between PC1 and **(a)** latitude and **(b)** longitude **(c)** Correlation between genetic (F_{ST}) and geographic distances estimated by Mantel test. The p-value remains significant after 999 replicates.

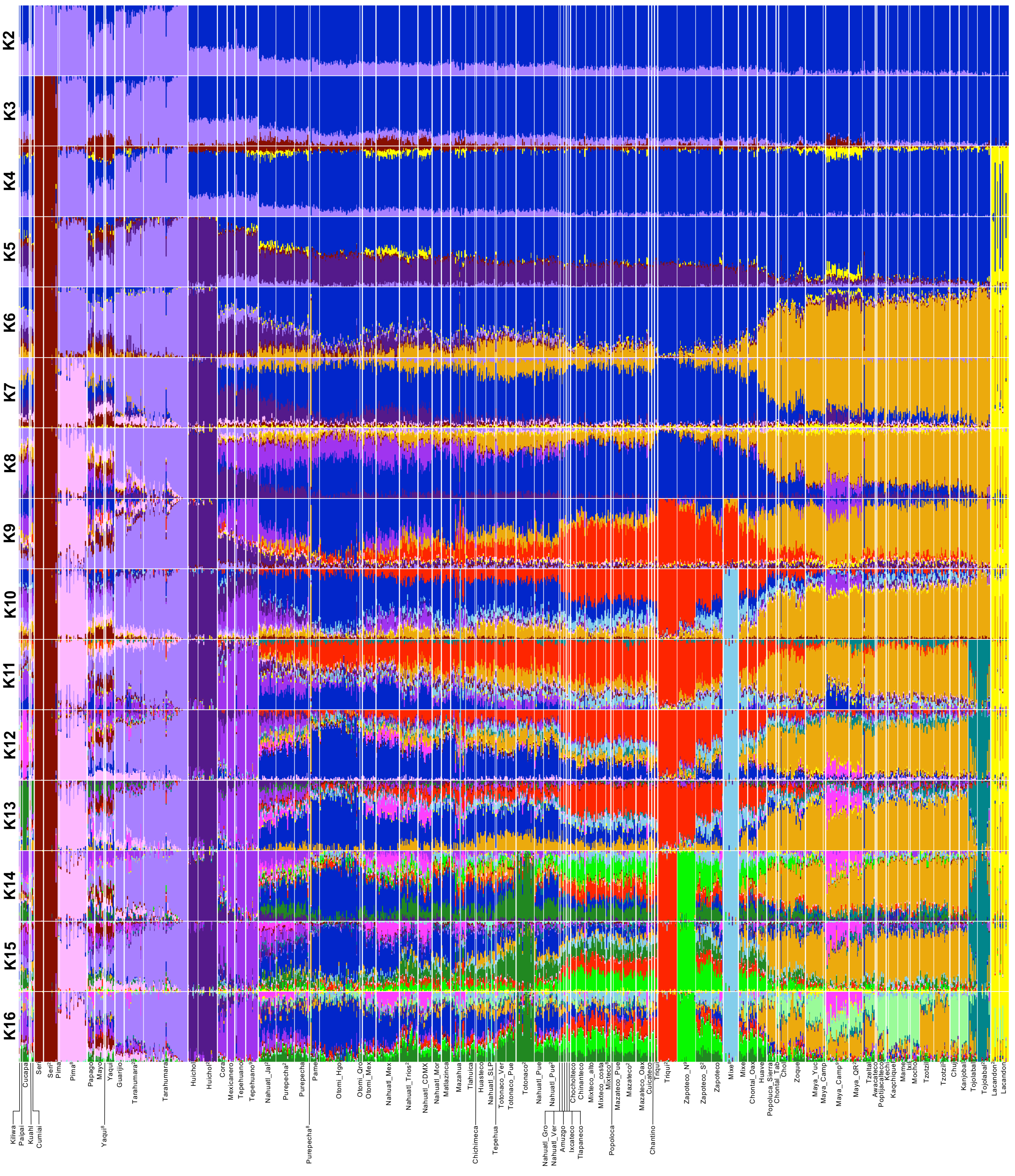

Supplementary Figure 4. Admixture analyses. Bar plots for each simulated cluster. Vertical bars represent one individual and y-axis represents the proportion of the genome assigned to each cluster (K = 2-16).

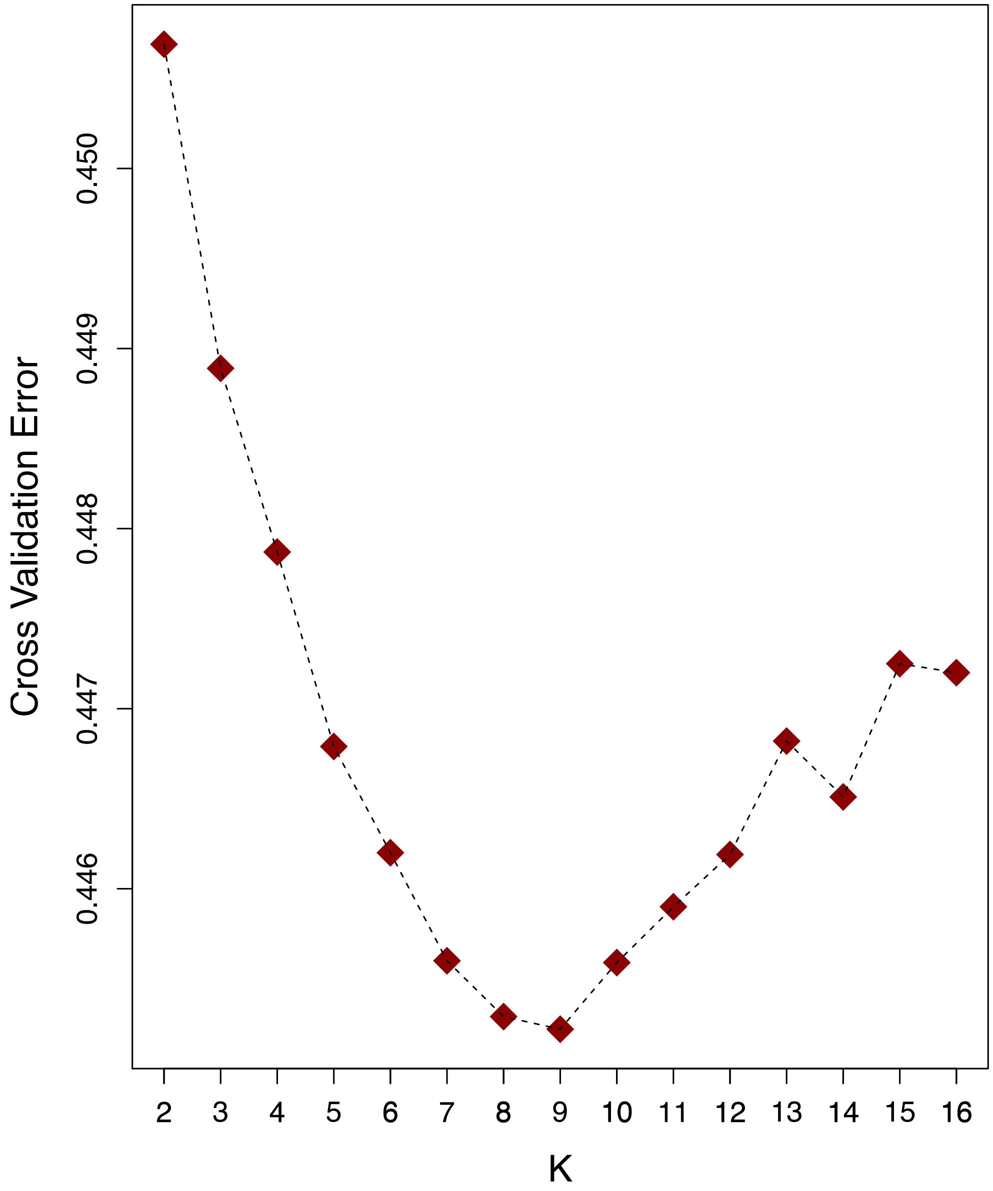

Supplementary Figure 5. cross-validation error for admixture analyses K = 2-16 clusters. Between each inferred cluster, the K = 9 had the lowest cross-validation error.

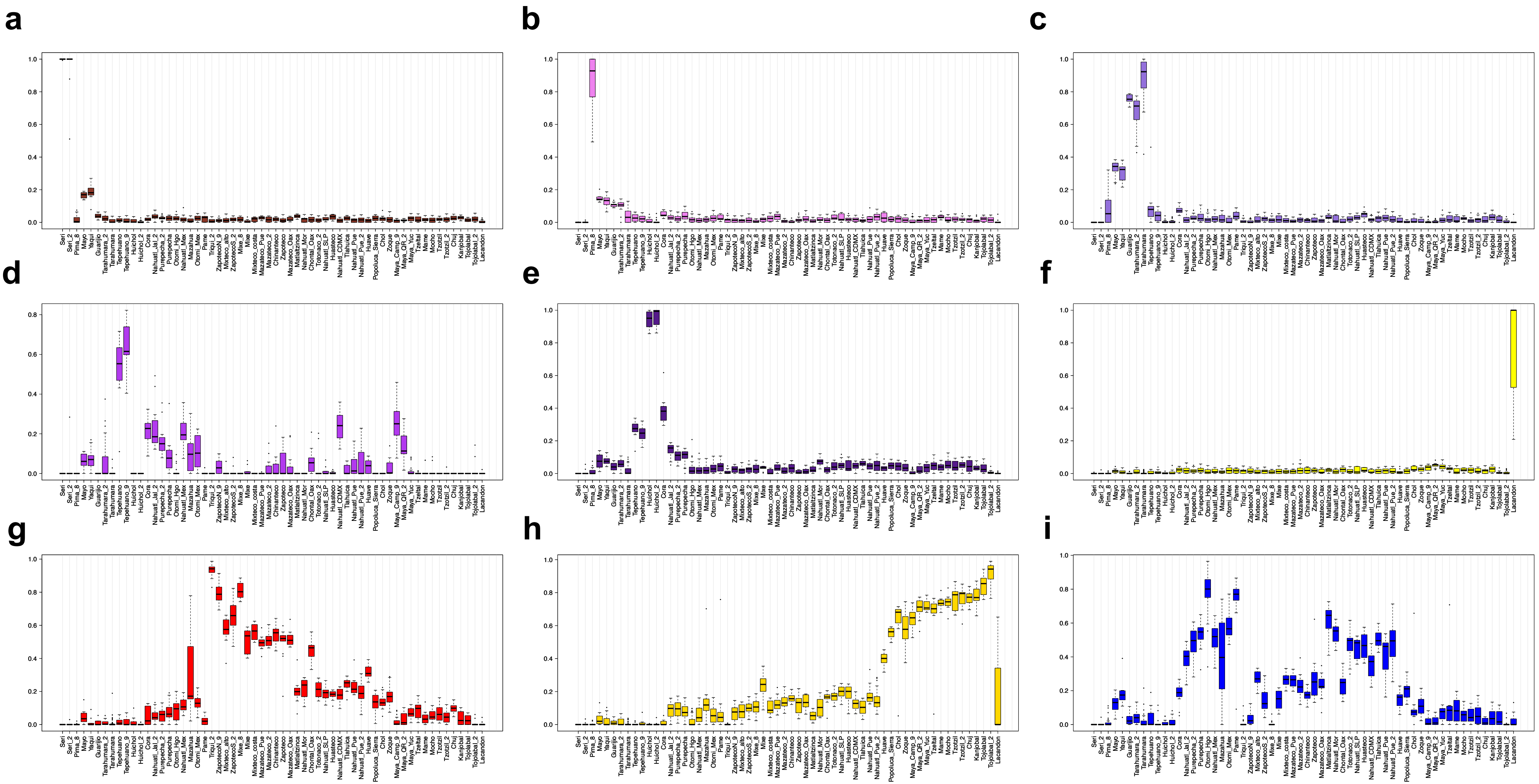

Supplementary Figure 6. Boxplots of each ancestral component identified by admixture (K=9) in each ethnic group. The graphs show the enrichment of some components in different ethnic groups (**(a to f)** and regions (**g to i).** We are only showing the populations with a n ≥ 10 (see supplementary table 1). In each boxplot the middle line is the median, the lower and upper hinges correspond to the first and third quartiles and whiskers the non-outlier range, while points are outliers.

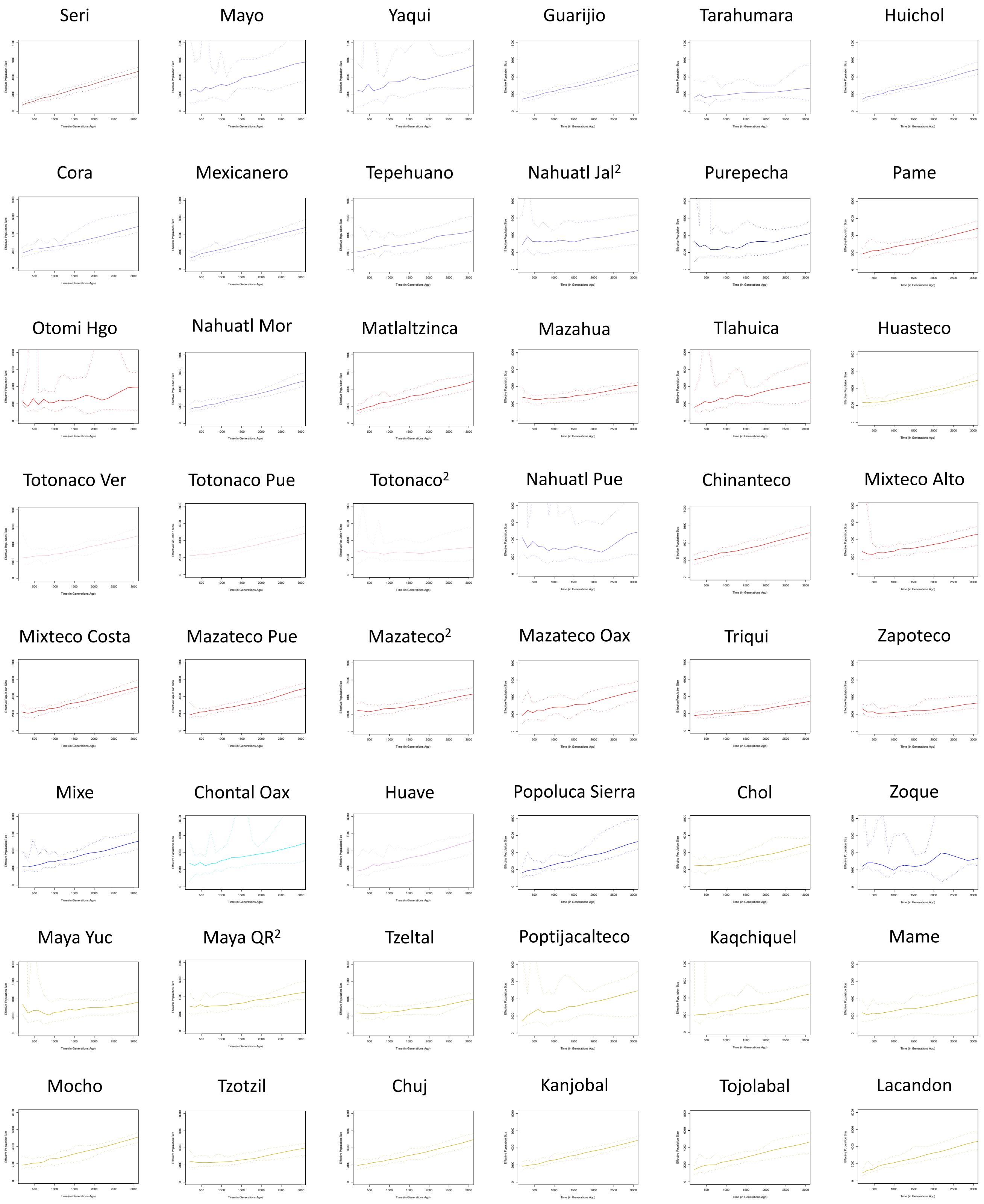

Supplementary Figure 7. Effective population size across time estimated from linkage disequilibrium in each tested population. Solid lines represent the effective population size and dashed lines represent the 95% confidence interval.

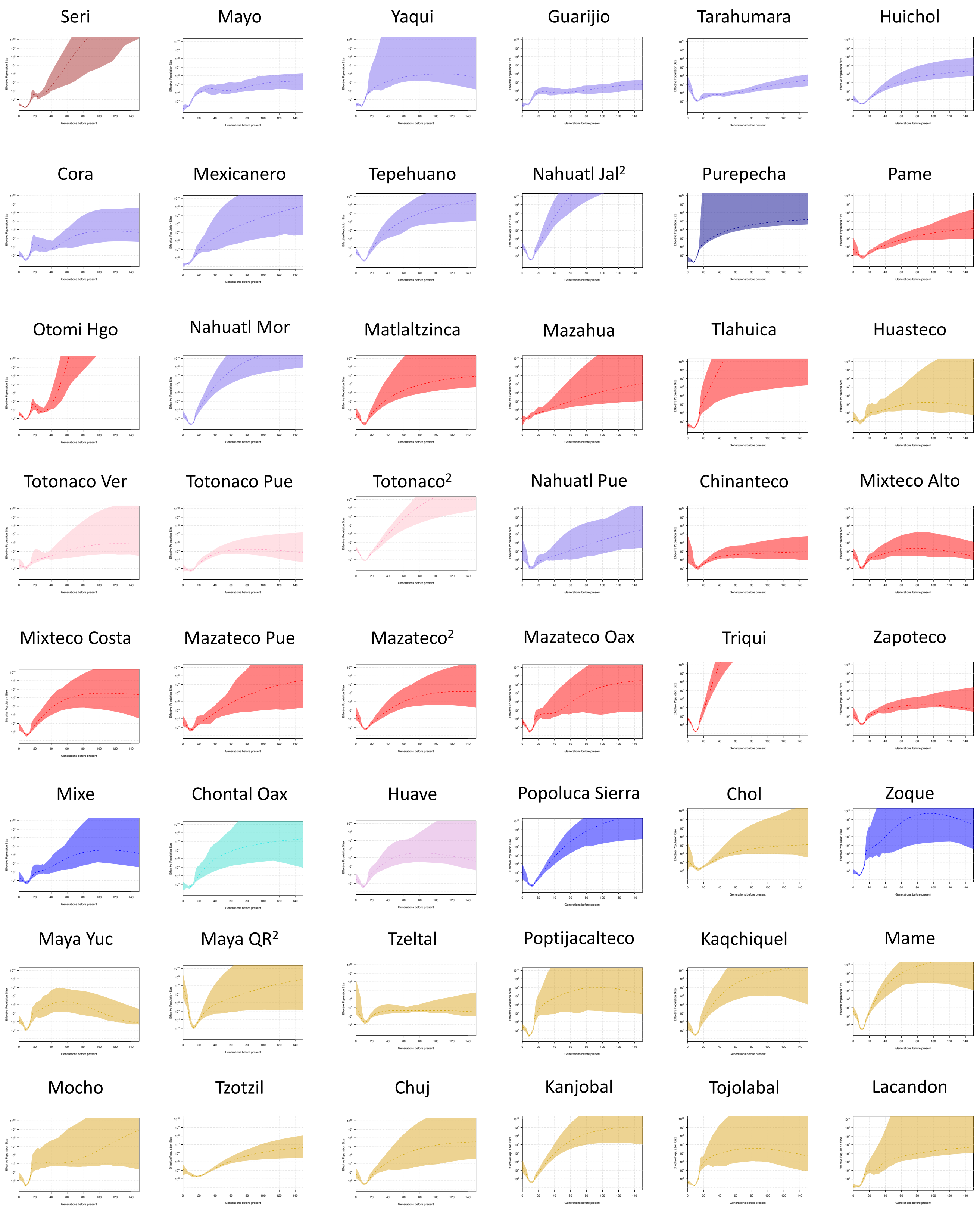

Supplementary Figure 8. Reconstruction of Ne across time based on IBD segments. In each one of the 48 populations examined here, there are evidence of a bottleneck spanning 15-30 generations ago. Dashed lines represent the effective populations size, colored regions represent the 95% confidence intervals.

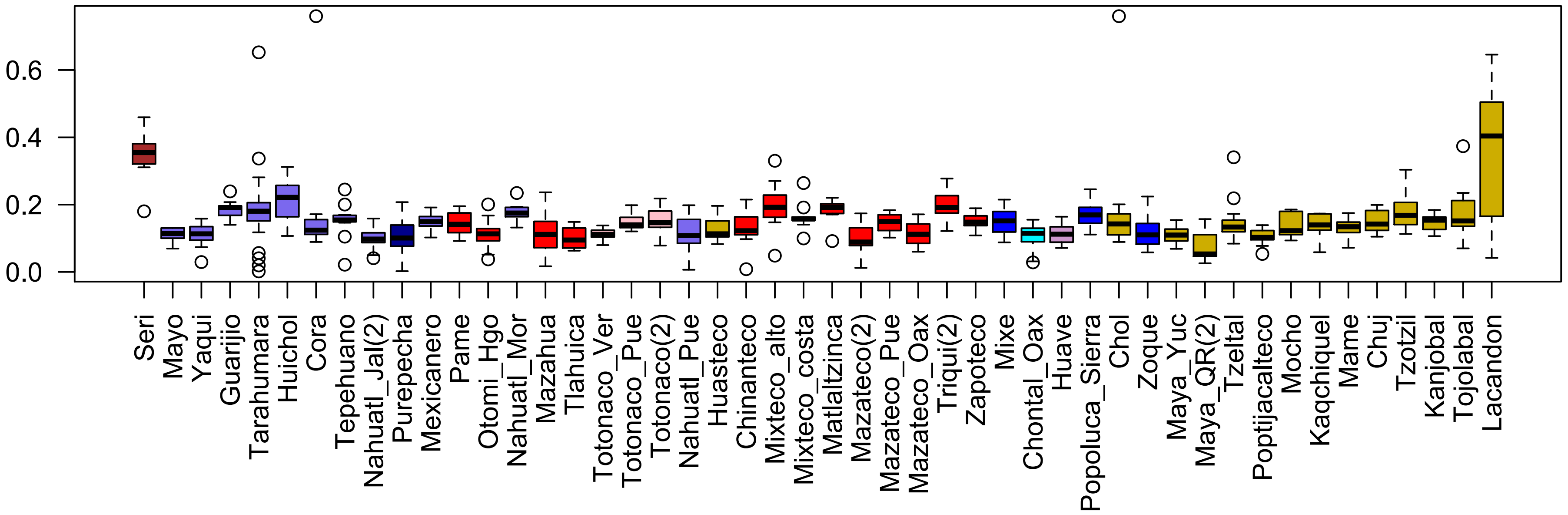

Supplementary Figure 9. Proportion of the genome in ROH in Indigenous groups of Mexico. We are showing the ROH proportion for 48 selected populations (see supplementary table 3). Boxplots shows the median (middle line), the first and third quartiles (lower and upper hinges) non-outlier range (whiskers) and individual points are the outliers.

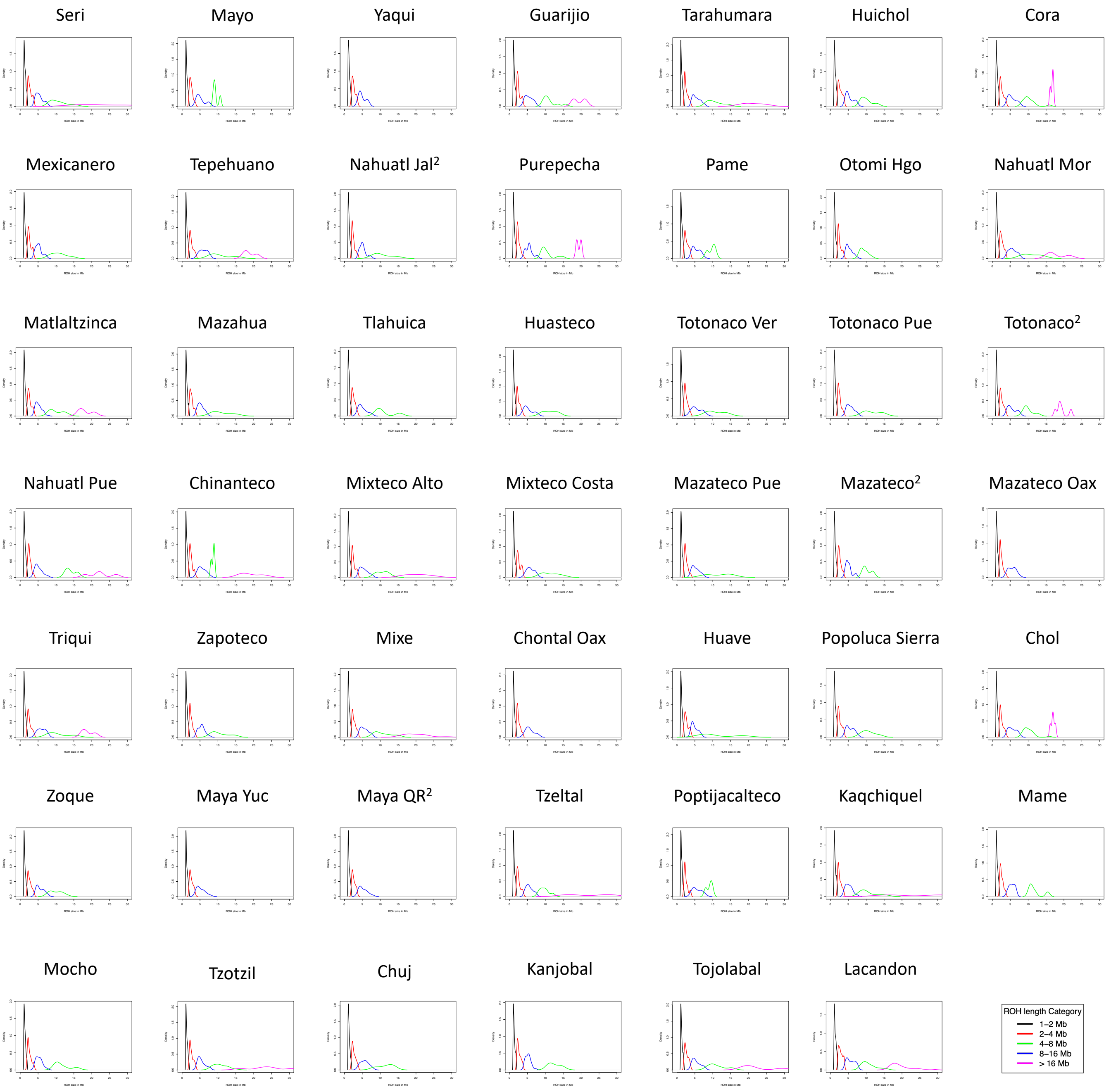

Supplementary Figure 10. Distribution of ROH in each population. Kernel distribution of ROH by length category expressed in Mb.

a

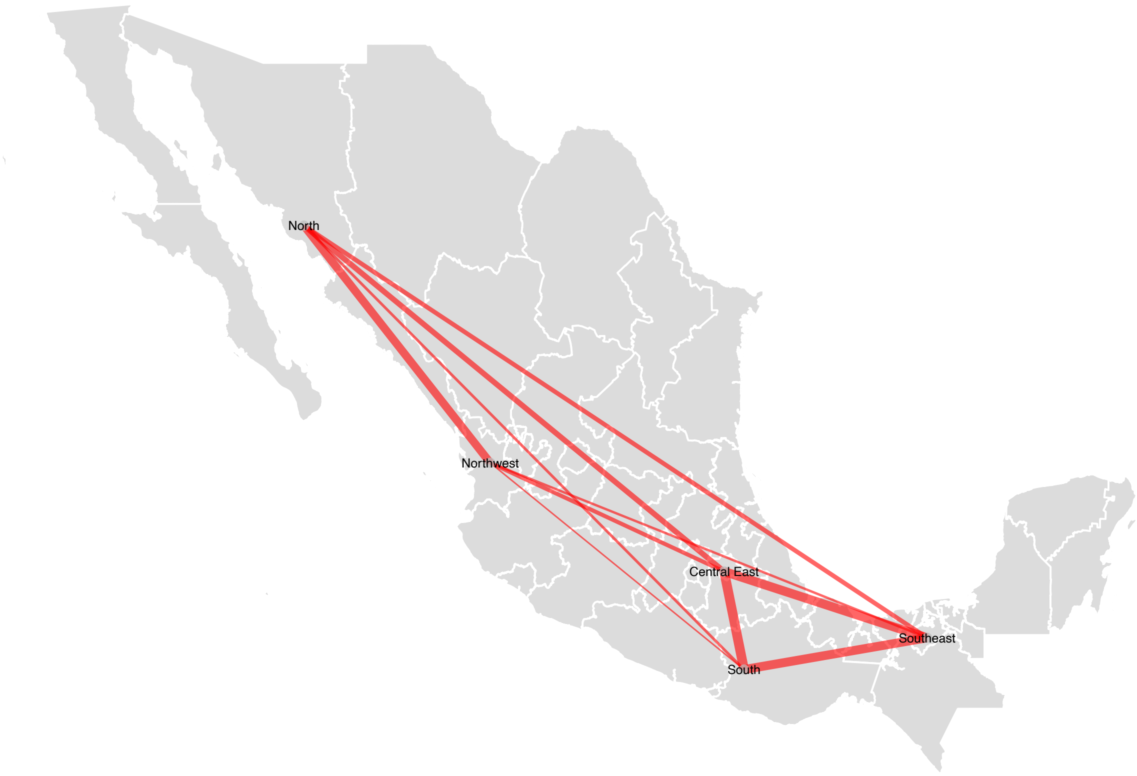

b

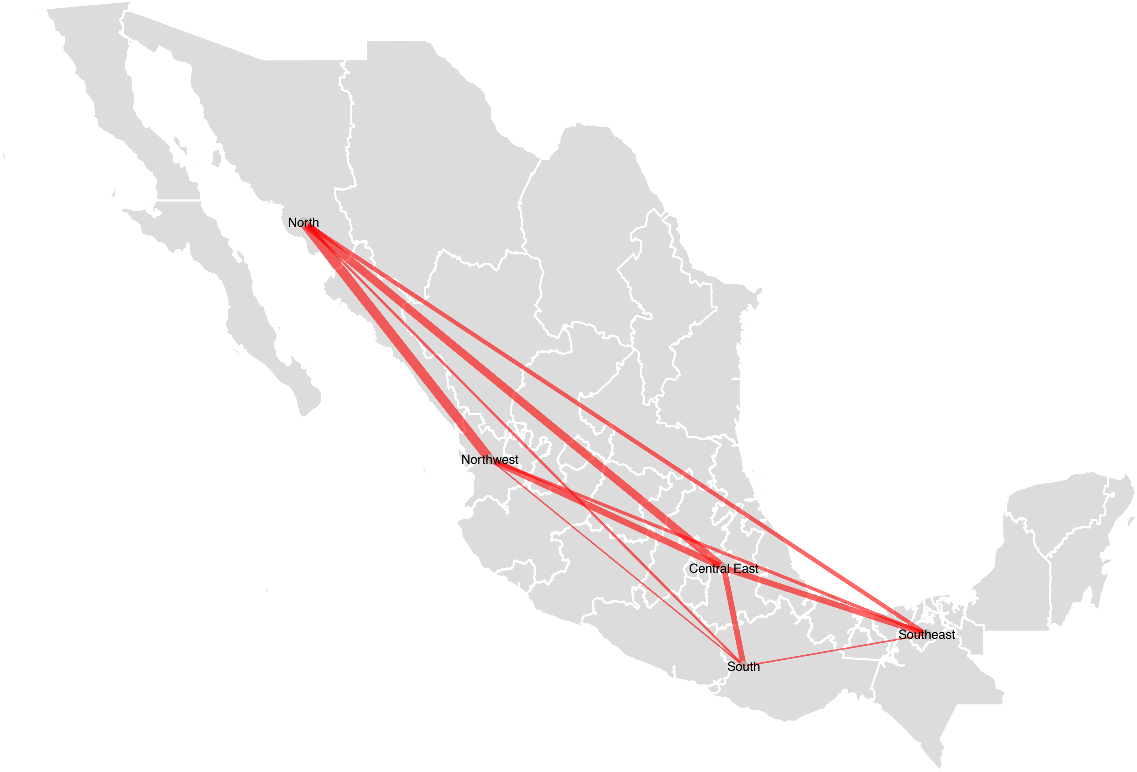

Supplementary Figure 11. IBD segments analysis performed in Indigenous populations clustered by geographic region. (a) IBD segments ranged from 5 -10 cM **(b)** IBD segments above 10 cM. The width of each edge is proportional to the mean IBD length. Made with Natural Earth. Free vector and raster map data @ naturalearthdata.com.

a

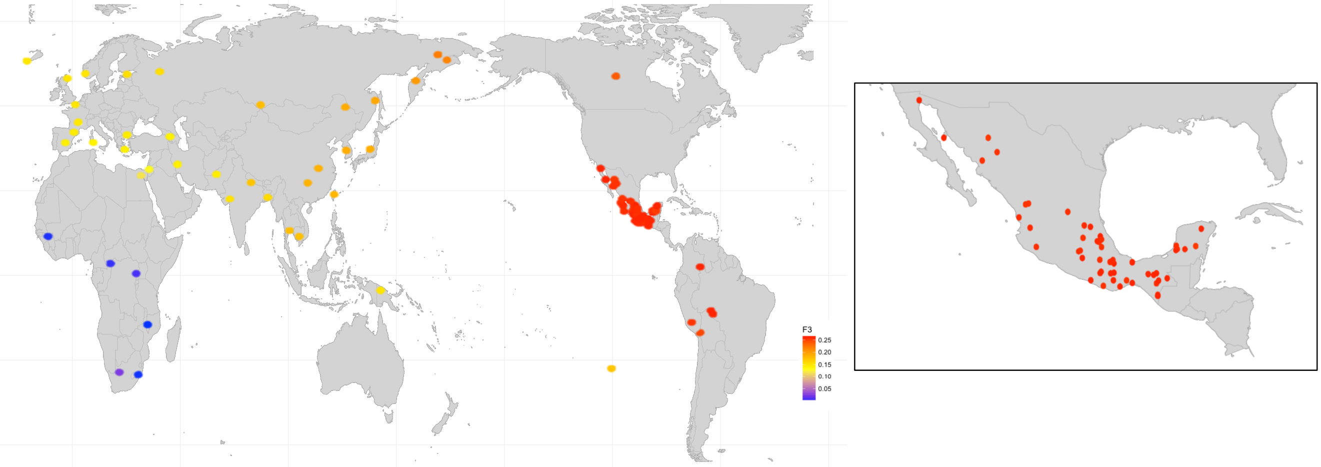

b

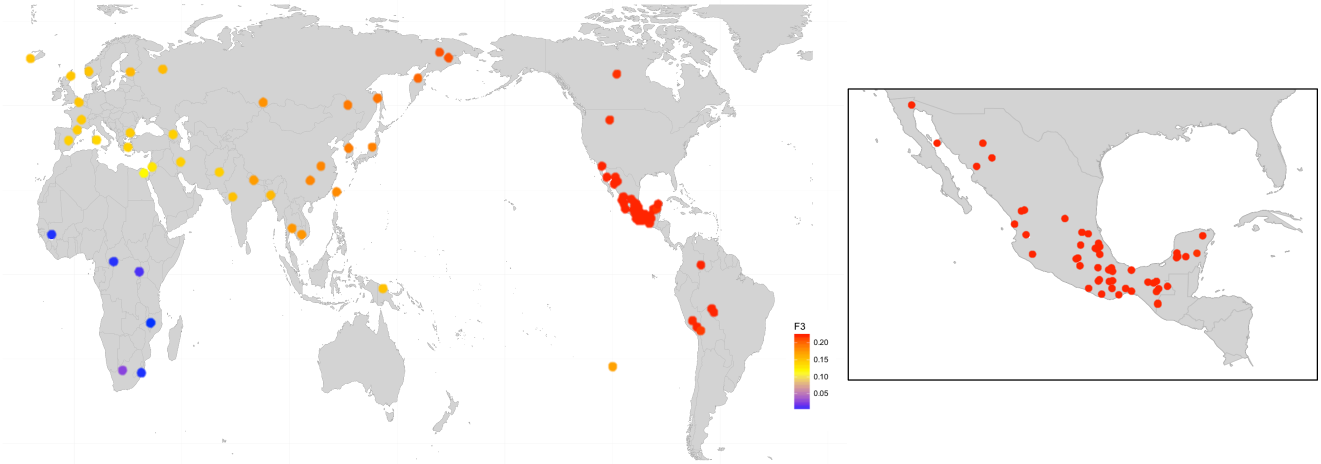

Supplementary Figure 12. F3 statistics in the form of *f3*(YRI; Ancient, Mexican Indigenous population). f3 statistic for **(a)** USR1 and **(b)** Anzick-1. Yoruba and Han populations were also included in this analysis (Supplementary Data 8). Made with Natural Earth. Free vector and raster map data @ naturalearthdata.com.

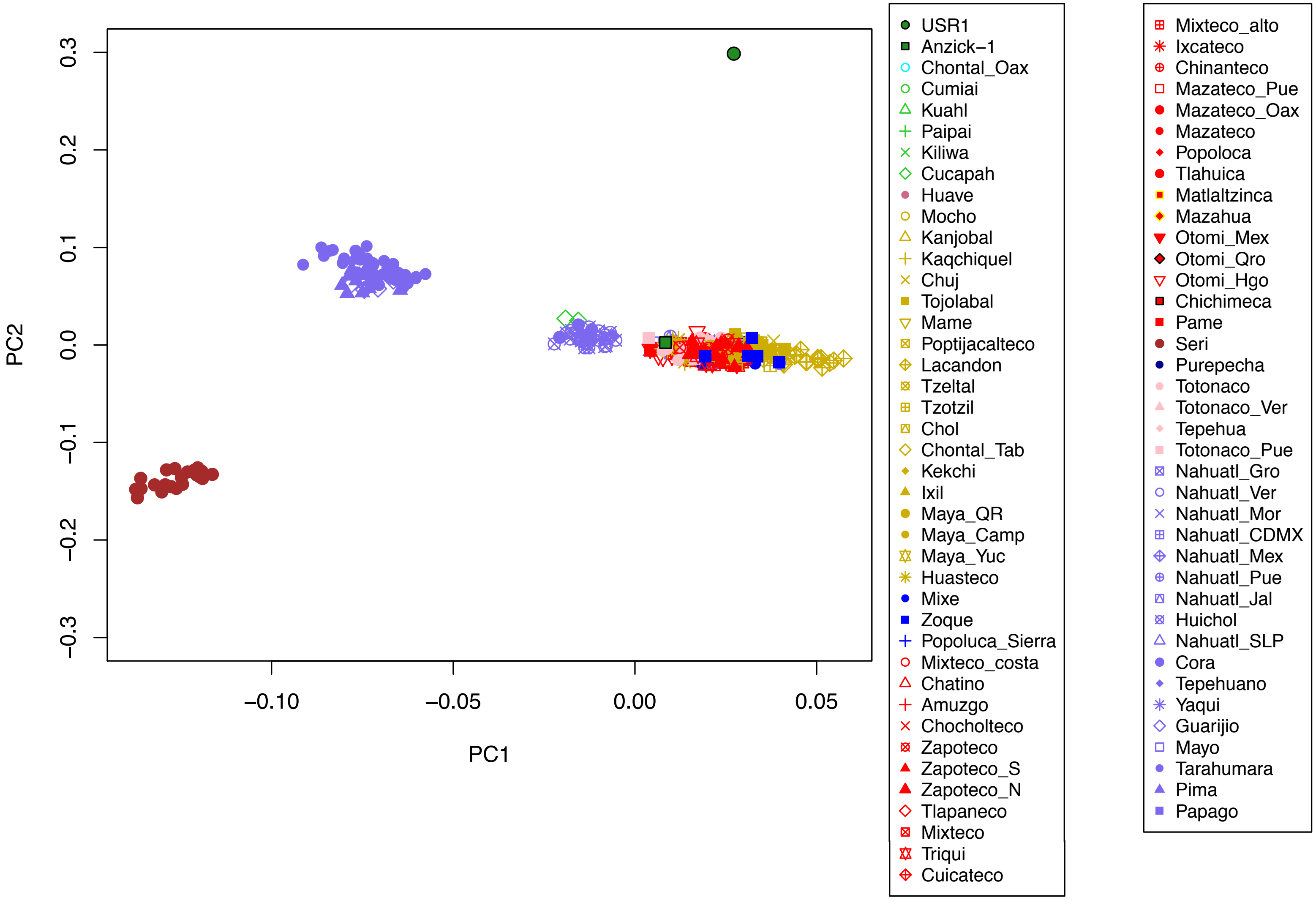

Supplementary Figure 13. PCA projection of Mexican Indigenous populations including ancient genomes from America. Top two PC projection shows that USR1 acts as outlier for all Indigenous populations from Mexico and that Mesoamerican populations are more closely related to ancient genome of Anzick-1. Except for USR1 and Anzick-1, colors represent the linguistic family according to figure 1.

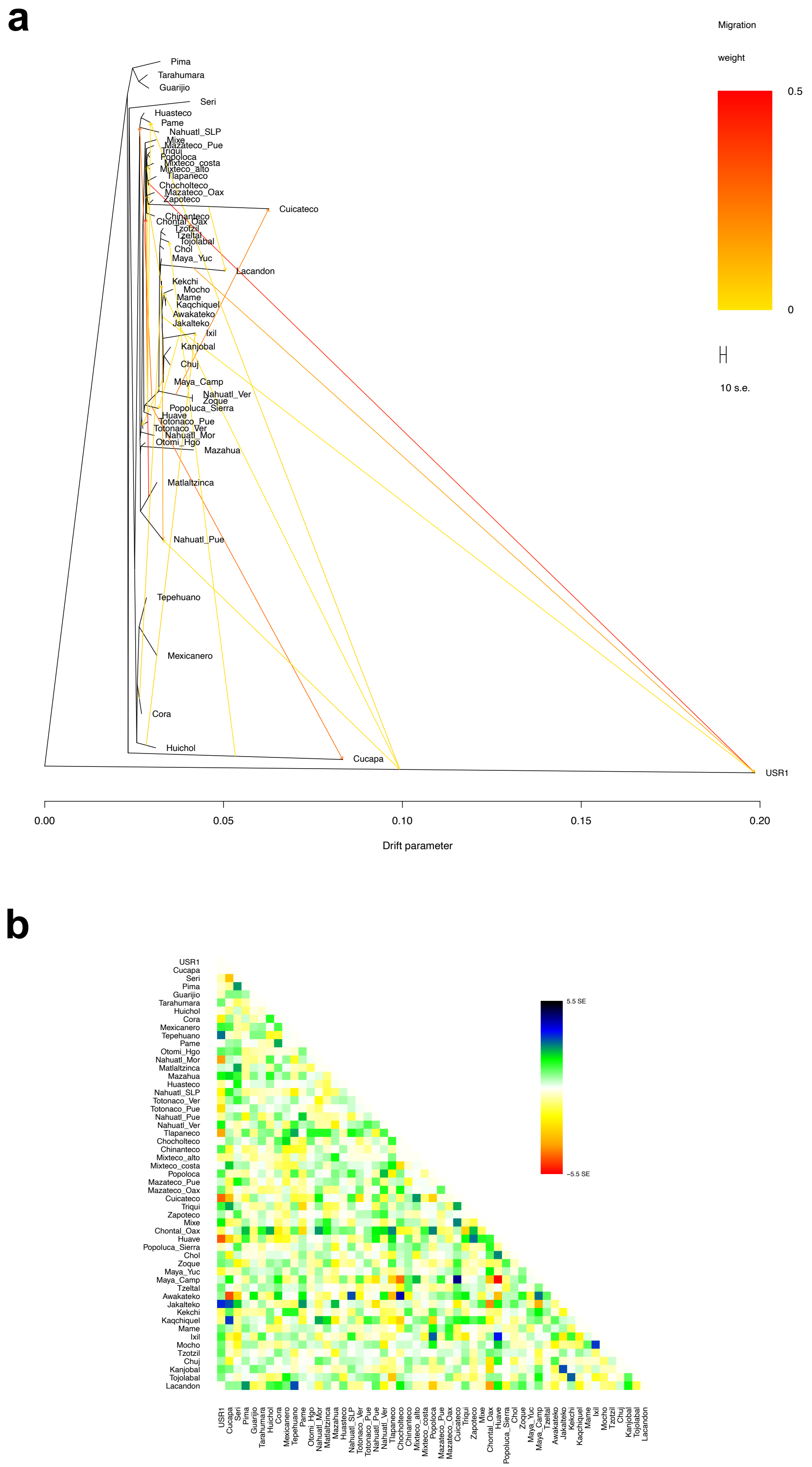

Supplementary Figure 14. Maximum likelihood Tree assuming 20 migrations performed in Treemix. **(a)** Maximum likelihood tree simulating 20 migration events shows a multiple migration waves between populations from Aridoamerica and Mesoamerica. **(b)** Residuals of the likelihood tree.

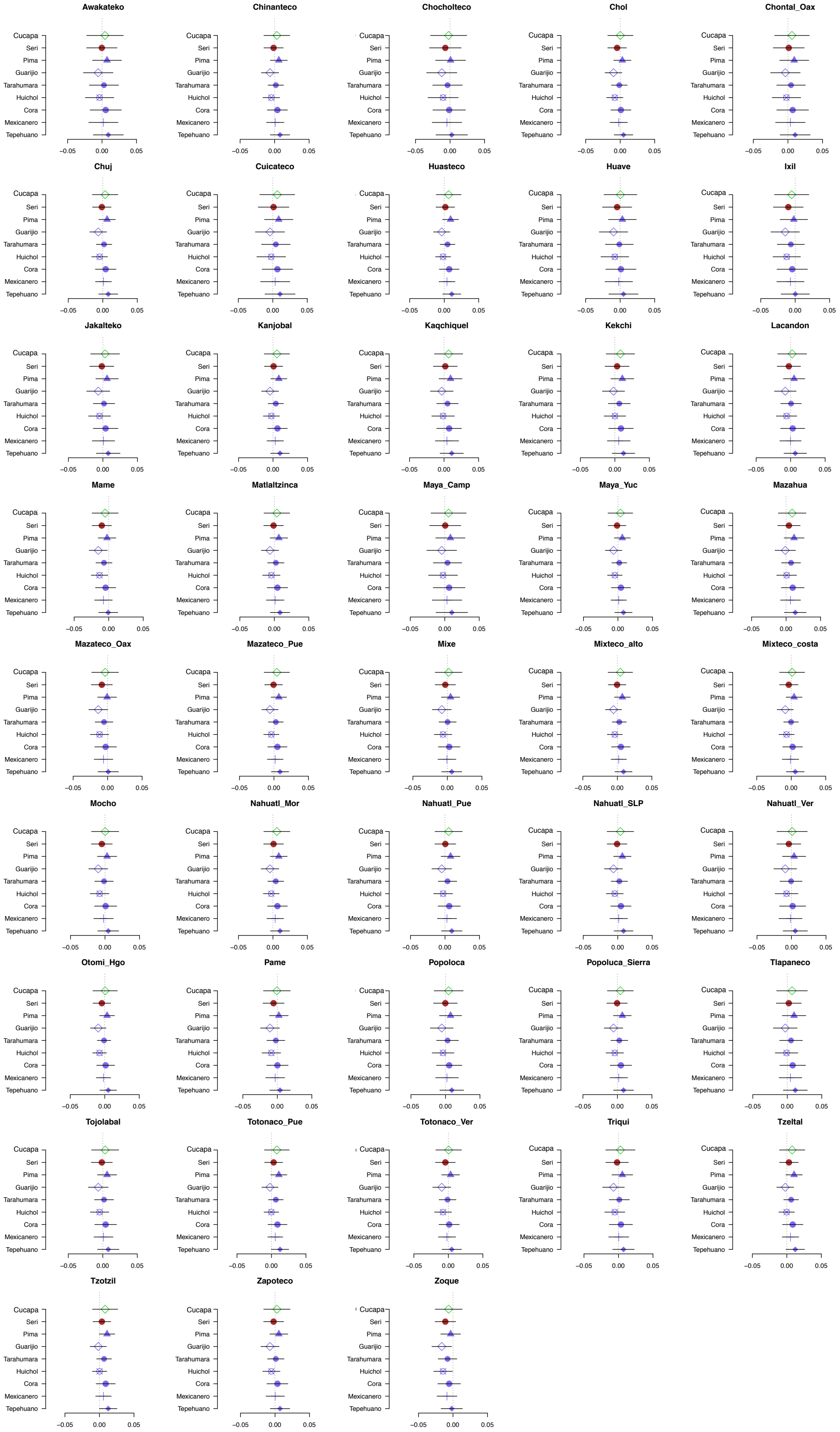

Supplementary Figure 15. D-statistic in the form of *D*(YRI, ANCB/NNA; AA, MA). We used the ancient Lucier individual as a representative of ANCB/NNA ancestry. Error bars represents 3 standard errors.

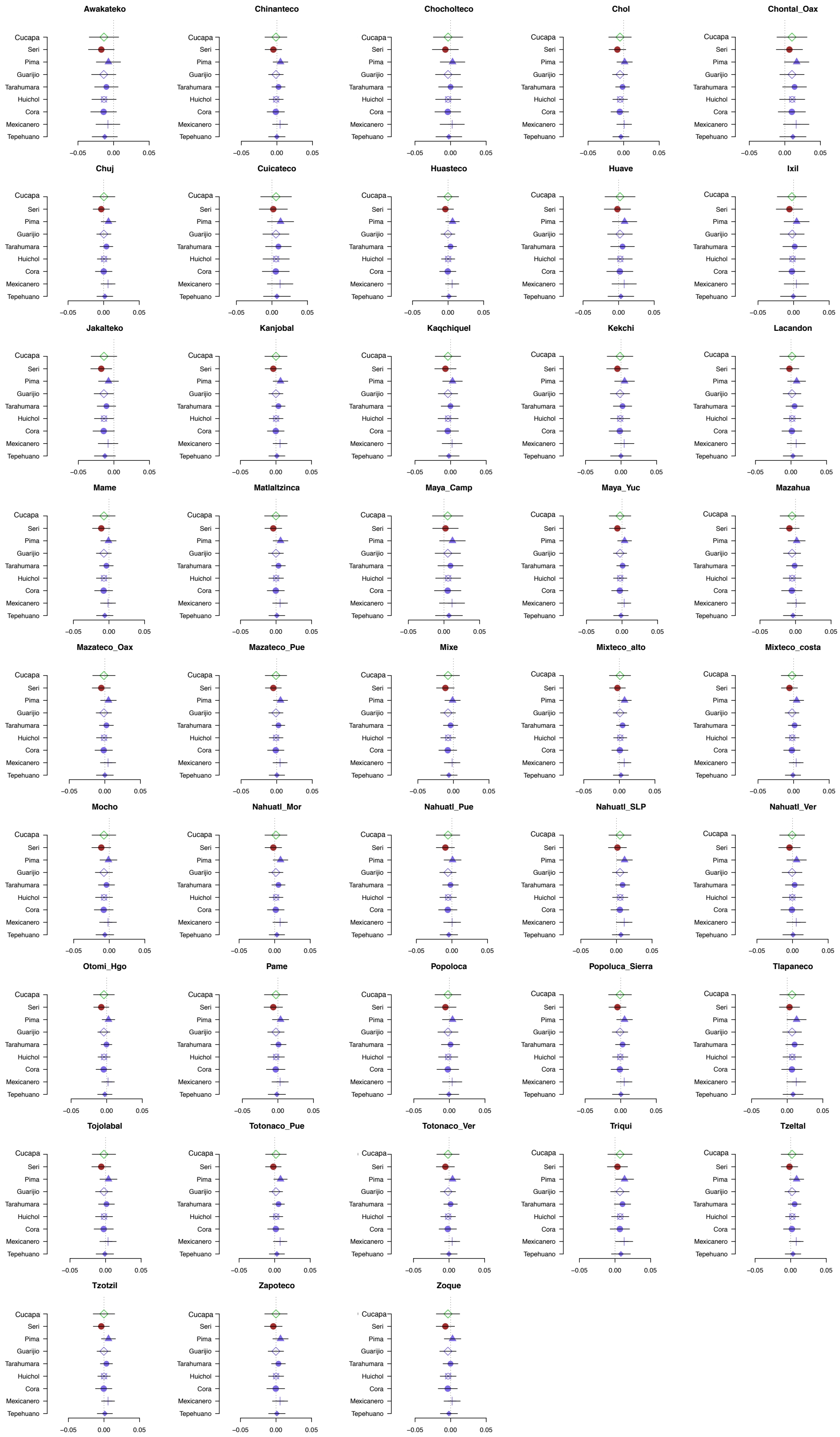

Supplementary Figure 16. D-statistic in the form of *D*(YRI, ANCB/NNA; AA, MA). We used the ancient Athabaskan individual as a representative of ANCB/NNA ancestry. Error bars represents 3 standard errors.

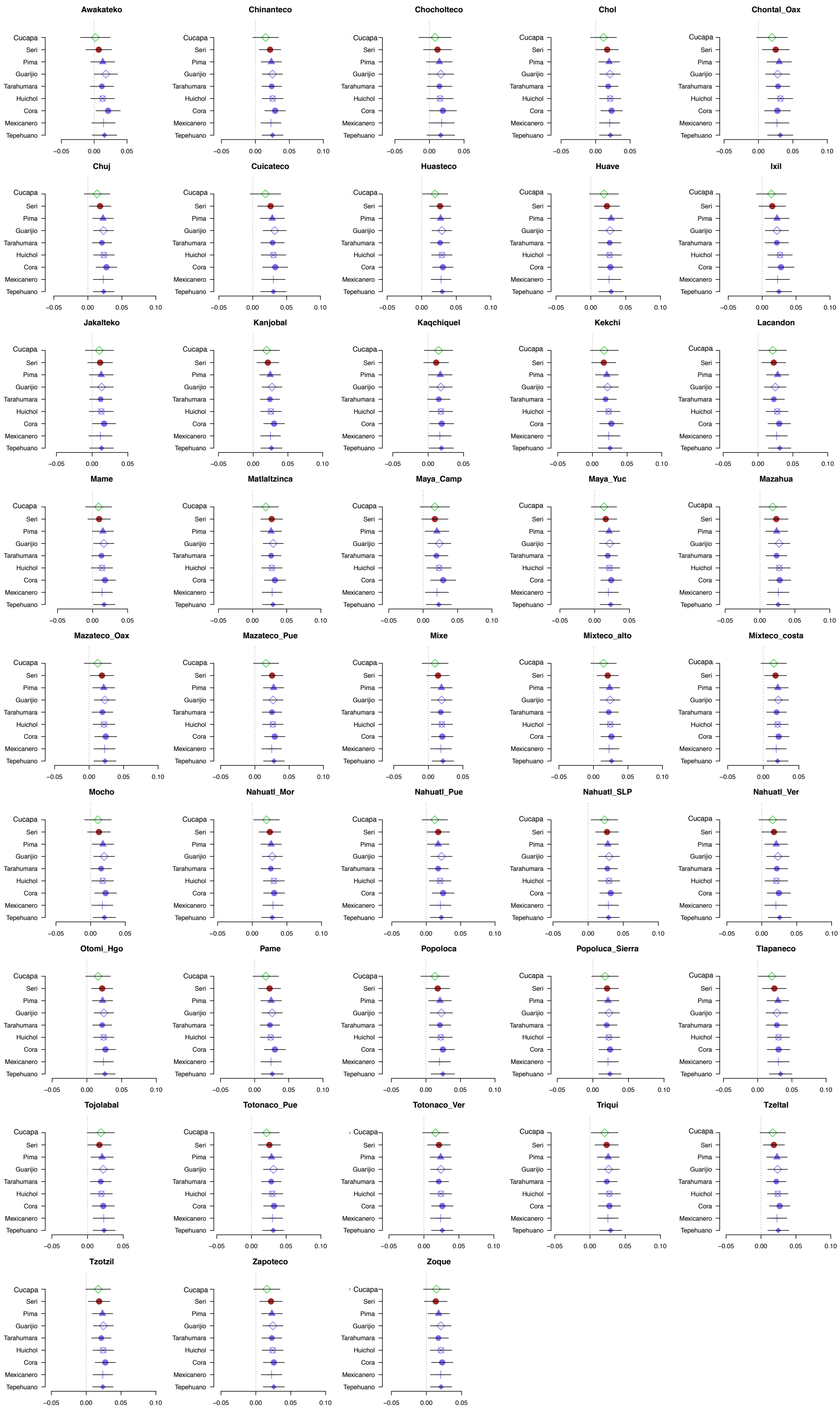

Supplementary Figure 17. D-statistic in the form of *D*(YRI, AA; Anzick-1, MA).
Error bars represent 3 standard errors.

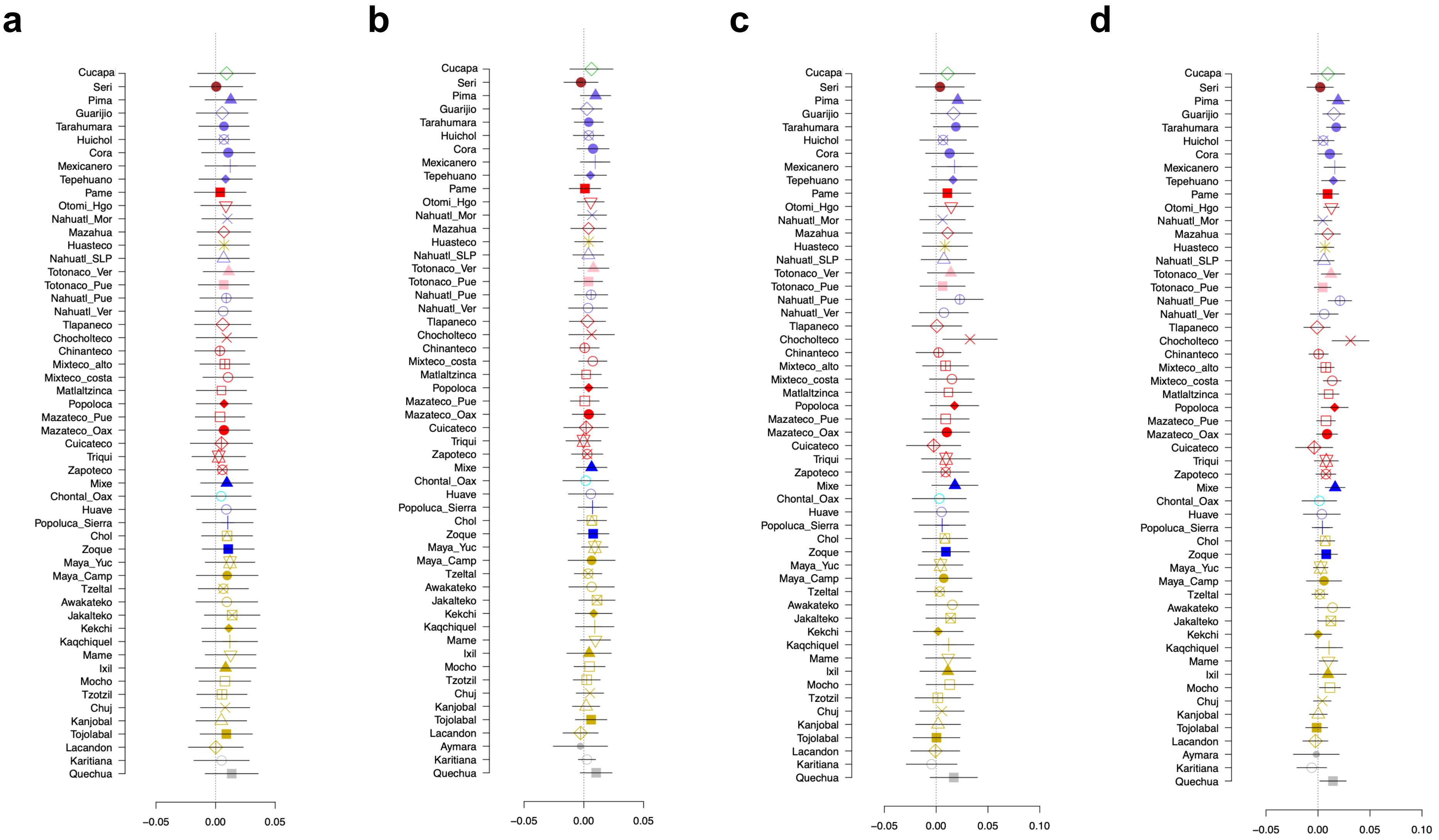

Supplementary Figure 18. D statistics comparing Mexican indigenous populations with ancient genomes of America. (a)D in the form of *D*(Yoruba, USR1; Mexican Native population, Aymara). **(b)** D in the form of *D*(Yoruba, USR1; Mexican Native population, Tzotzil). **(c)** D in the form of *D*(Yoruba, Anzick-1; Mexican Native population, Aymara). **(d)** D in the form of *D*(Yoruba, Anzick-1; Mexican Native population, Tzotzil). In **a** to **d**, error bars represent 3 standard errors.
