# Supplementary material for: The genomic landscape of Mexican Indigenous populations brings insights into the peopling of the Americas
Source: Nat Commun. 2021 Oct 12;12:5942. doi: 10.1038/s41467-021-26188-w (PMC8511047; doi:10.1038/s41467-021-26188-w)
Supplement: Supplementary file 3 — Description of Additional Supplementary Files [file 41467_2021_26188_MOESM3_ESM.pdf]

Supplementary Data Legends NCOMMS-19-19512C

Supplementary Data 1. F_{ST} values by pairs of populations.

Supplementary Data 2. Divergence time between pairs of population expressed in Ka.

Supplementary Data 3. IBD shared segments above 7 cM between Mexican Indigenous populations from the North and the rest of the regions.

Supplementary Data 4. IBD shared segments above 7 cM between Mexican Indigenous populations from the Northwest and the rest of the regions.

Supplementary Data 5. IBD shared segments above 7 cM between Mexican Indigenous populations from the Center and the rest of the regions.

Supplementary Data 6. IBD shared segments above 7 cM between Mexican Indigenous populations from the South and the rest of the regions.

Supplementary Data 7. IBD shared segments above 7 cM between Mexican Indigenous populations from the Southeast and the rest of the regions.

Supplementary Data 8. Otuput f3 statistics in the form of *f3*(ancient genome, tested population; Yoruba).
