# Supplementary material for: The genomic landscape of Mexican Indigenous populations brings insights into the peopling of the Americas
Source: Nat Commun. 2021 Oct 12;12:5942. doi: 10.1038/s41467-021-26188-w (PMC8511047; doi:10.1038/s41467-021-26188-w)
Supplement: Supplementary file 5 — Reporting Summary [file 41467_2021_26188_MOESM5_ESM.pdf]

nature research

Corresponding author(s): Humberto García-Ortiz
Lorena Orozco

Last updated by author(s): Aug 27, 2021

Reporting Summary

Nature Research wishes to improve the reproducibility of the work that we publish. This form provides structure for consistency and transparency in reporting. For further information on Nature Research policies, see Authors & Referees and the Editorial Policy Checklist .

Statistics

For all statistical analyses, confirm that the following items are present in the figure legend, table legend, main text, or Methods section.

n/a	Confirmed	
☐	☒	The exact sample size (*n*) for each experimental group/condition, given as a discrete number and unit of measurement
☒	☐	A statement on whether measurements were taken from distinct samples or whether the same sample was measured repeatedly
☐	☒	The statistical test(s) used AND whether they are one- or two-sided<br>*Only common tests should be described solely by name; describe more complex techniques in the Methods section.*
☒	☐	A description of all covariates tested
☐	☒	A description of any assumptions or corrections, such as tests of normality and adjustment for multiple comparisons
☐	☒	A full description of the statistical parameters including central tendency (e.g. means) or other basic estimates (e.g. regression coefficient) AND variation (e.g. standard deviation) or associated estimates of uncertainty (e.g. confidence intervals)
☐	☒	For null hypothesis testing, the test statistic (e.g. *F*, *t*, *r*) with confidence intervals, effect sizes, degrees of freedom and *P* value noted<br>*Give P values as exact values whenever suitable.*
☒	☐	For Bayesian analysis, information on the choice of priors and Markov chain Monte Carlo settings
☐	☒	For hierarchical and complex designs, identification of the appropriate level for tests and full reporting of outcomes
☐	☒	Estimates of effect sizes (e.g. Cohen's *d*, Pearson's *r*), indicating how they were calculated

Our web collection on statistics for biologists contains articles on many of the points above.

Software and code

Policy information about availability of computer code

Data collection

Plink v1.9
Eigensoft v5.0.
SHAPEIT v2.17
RFMix v2
AdapterRemoval v2.1
bwa aln v0.6.2-r126
picard-tools v2.0.1
GATK v4.0
VCFtools v0.01.16
admixture v1.3
R v3.4
APE v5.1
DendroPy v4.0.0
adegenet v2.1.1
NeON v1.0
Beagle v5.1
IBDseq r1206
IBDNe 19Sep19.268
ggnetworkmap from the R package GGally v2.1.2
Admixtools v4.1
TreeMix v1.13
Hap-IBD v1.0

Data analysis | All data analyses in this work were done following the standard pipelines available for each software.

For manuscripts utilizing custom algorithms or software that are central to the research but not yet described in published literature, software must be made available to editors/reviewers. We strongly encourage code deposition in a community repository (e.g. GitHub). See the Nature Research guidelines for submitting code & software for further information.

Data

Policy information about availability of data

All manuscripts must include a data availability statement. This statement should provide the following information, where applicable:

- Accession codes, unique identifiers, or web links for publicly available datasets
- A list of figures that have associated raw data
- A description of any restrictions on data availability

The whole genotype data from the 716 Indigenous individuals from MAIS cohort is available under restricted access to protect the privacy of the participants and in alignment with the Institutional Review Board approval and the individual informed consents forms. Access can be obtained through a data-access agreement.

Field-specific reporting

Please select the one below that is the best fit for your research. If you are not sure, read the appropriate sections before making your selection.

☒ Life sciences ☐ Behavioural & social sciences ☐ Ecological, evolutionary & environmental sciences

For a reference copy of the document with all sections, see nature.com/documents/nr-reporting-summary-flat.pdf

Life sciences study design

All studies must disclose on these points even when the disclosure is negative.

Sample size	No statistical methods were used to predetermine sample sizes. Sample size in this study was limited by the aviability of samples from each ethnic group included here.
Data exclusions	Genome wide genotyping was done for all samples in this work. The following paragraph explaining the exclusion procceses is included in the methods subsections "Samples and data handling": "To perform our estimations, we generated several datasets merging our genotype data with those previously published for several world-wide populations and modern and ancient Native American individuals as follows. For data generated using only an SNV array, we performed the data handling and quality control procedures in Plink V 1.9(ref.62). Each dataset was processed individually, including per marker and per sample examinations. We removed SNVs with genotyping rates < 98% and those with a minor allele frequency of 1%, and then removed mitochondrial and sex chromosome SNVs. Finally, we excluded individuals with missing rates > 3% and with discordant gender information."
Replication	Due the nature of our population genetics study, there is no need for replication because we did not perform any experimental procedure.
Randomization	Samples were grouped based on the ethnic group who each participant report. In other cases were grouped based on their geographic location (North, Northwest, Center, South and Southeast).
Blinding	No blinding techniques were implemented because they are not necesary for population genetics studies.

Reporting for specific materials, systems and methods

We require information from authors about some types of materials, experimental systems and methods used in many studies. Here, indicate whether each material, system or method listed is relevant to your study. If you are not sure if a list item applies to your research, read the appropriate section before selecting a response.

Materials & experimental systems

n/a	Involved in the study	
☒	☐	Antibodies
☒	☐	Eukaryotic cell lines
☒	☐	Palaeontology
☒	☐	Animals and other organisms
☐	☒	Human research participants
☒	☐	Clinical data

Methods

n/a	Involved in the study	
☒	☐	ChIP-seq
☒	☐	Flow cytometry
☒	☐	MRI-based neuroimaging

Human research participants

Policy information about studies involving human research participants

Population characteristics	The study population is comprised by Mexican Indigenous volunteers older than 18 years old. Ethnic group affiliation and geographic location are the only relevant covariates used in this study.
Recruitment	The MAIS cohort was collected between 2011-2015. All volunteers were recruited in their own communities. Over the sampling procedure, no self-selection bias was introduced due to the recruitment was addressed to the general population.
Ethics oversight	This study was designed in accordance with the Declaration of Helsinki and approved by the Research, Ethics, and Biosafety Human Committees of the Instituto Nacional de Medicina Genómica (INMEGEN) in Mexico City (protocol number 31/2011/I) with the support of the National Commission for the Development of Indigenous Communities (CDI, from the Spanish Comisión Nacional para el Desarrollo de Pueblos Indígenas) and with the agreement of the Indigenous leaders from each community. All participants provided written informed consent, and authorities or community leaders participated as translators when necessary.

Note that full information on the approval of the study protocol must also be provided in the manuscript.
